# Assessment of Radiation Attenuation Properties in Dental Implants Using Monte Carlo Method †

**DOI:** 10.3390/bioengineering12070762

**Published:** 2025-07-14

**Authors:** Ali Rasat, Selmi Tunc, Yigit Ali Uncu, Hasan Ozdogan

**Affiliations:** 1 Department of Maxillofacial Radiology, Faculty of Dentistry, Akdeniz University, 07058 Antalya, Turkey; alicanras07@hotmail.com; 2 Department of Biomedical Equipment Technology, Vocational School of Technical Sciences, Akdeniz University, 07070 Antalya, Turkey; yuncu@akdeniz.edu.tr; 3 Department of Medical Imaging Techniques, Vocational School of Health Services, Antalya Bilim University, 07190 Antalya, Turkey; hasan.ozdogan@antalya.edu.tr

**Keywords:** dental implant, dental radiology, Monte Carlo simulation, radiation attenuation

## Abstract

This study investigated the radiation attenuation characteristics of commonly used dental implant materials across an energy spectrum relevant to dental radiology. Two titanium implants were examined, with densities of 4.428 g/cm^3^ and 4.51 g/cm^3^, respectively. The first consisted of 90.39% titanium, 5.40% aluminum, and 4.21% vanadium, while the second comprised 58% titanium, 33% oxygen, 7% iron, 1% carbon, and 1% nitrogen. The third material was a zirconia implant (5Y form) composed of 94.75% zirconium dioxide, 5.00% yttrium oxide, and 0.25% aluminum oxide, exhibiting a higher density of 6.05 g/cm^3^. Monte Carlo simulations (MCNP6) and XCOM data were utilized to estimate photon source parameters, geometric configuration, and interactions with biological materials to calculate the half-value layer, mean free path, and tenth-value layer at varying photon energies. The results indicated that titanium alloys are well suited for low-energy imaging modalities such as CBCT and panoramic radiography due to their reduced artifact production. While zirconia implants demonstrated superior attenuation at higher energies (e.g., CT), their higher density may induce beam-hardening artifacts in low-energy systems. Future research should validate these simulation results through in vitro and clinical imaging and further explore the correlation between material-specific attenuation and CBCT image artifacts.

## 1. Introduction

A dental implant is a screw-shaped post made of biocompatible materials, such as titanium or zirconium, surgically inserted into the jawbone to support a prosthetic tooth or dental bridge [1,2]. Dental implants represent a widely accepted and effective treatment option for individuals who have lost teeth due to trauma, disease, or other causes. The initial exploration of dental implants began in the 1960s, with the pivotal discovery that pure titanium could achieve direct integration with bone, a breakthrough that transformed the field of implant dentistry [3]. This phenomenon, known as osseointegration, forms the biological foundation of oral implantology and is characterized by direct bone-to-implant contact. Mechanical stability is further enhanced by bone ingrowth into the micro-rough surface of the implant [4].

Over the next two decades, various materials and implant designs underwent clinical evaluation, including aluminum oxide-based ceramic implants [5], titanium plasma-sprayed surfaces, and titanium–aluminum–vanadium alloys [6]. By the late 1980s, commercially pure titanium became the standard material for dental implants due to its biocompatibility and clinical success [7].

Despite their advantages, titanium implants present esthetic limitations, particularly the discoloration of peri-implant soft tissues in patients with a thin gingival biotype. To overcome these concerns, zirconium dioxide (ZrO_2_) implants were developed, offering improved biocompatibility, reduced bacterial adhesion, and superior esthetic outcomes compared to titanium [8]. However, their clinical use remains limited due to higher costs and reduced availability relative to titanium implants [9].

Common imaging modalities in implant radiology include intraoral radiography, panoramic radiography, computed tomography (CT), and cone-beam computed tomography (CBCT) [10,11]. In CT and CBCT scans, high-density materials such as metal implants preferentially absorb low-energy X-rays, effectively increasing the average energy of the beam that reaches the detector. This phenomenon, known as beam hardening, leads to reconstruction artifacts, typically black bands and white streaks that compromise image quality, prolong interpretation time, and hinder diagnostic accuracy by obscuring critical anatomical structures [12,13]. Radiological procedures depend on the transmission, absorption, and scattering of radiation by tissues and anatomical structures [14]. Accurate estimation of tissue thickness in the radiation path and careful selection of the radiation source are essential to minimize patient exposure and optimize imaging outcomes. Reliable data on beam interactions, particularly under narrow-beam geometries, are crucial in fields such as diagnostic radiology, nuclear medicine, and radiotherapy. Understanding phenomena like beam hardening can help improve image quality while reducing radiation dose [15]. The literature includes studies that measure parameters such as half-value layer (HVL), mean free path (MFP), tenth-value layer (TVL), and attenuation coefficients using various materials and experimental methodologies [16,17,18,19,20].

The Monte Carlo (MC) method is a numerical technique that employs random sampling and probabilistic modeling to solve complex problems [21]. It is widely utilized across scientific and engineering disciplines for statistical and numerical analyses [22]. MC simulation represents natural processes through direct simulation of all possible outcomes, replacing factors with inherent uncertainty by assigning them probability distributions [23,24]. Today, it is a well-established tool for addressing analytically intractable problems and scenarios where experimental investigation is time-consuming, costly, or impractical. Researchers apply MC methods to study complex systems, explore variables that are difficult to measure experimentally, and conduct repeatable and modifiable simulations [25]. By using probability distributions and random number generation, the method enables estimation of physical or mathematical averages that are otherwise difficult to calculate analytically or numerically [26]. In the context of X-ray dosimetry, MC simulations track the trajectories of individual photons as they exit the X-ray source, interact with the simulated anatomical model, and undergo scattering and absorption events. The energy deposited at each interaction site is calculated to estimate dose distribution [27].

In this study, the Monte Carlo N-Particle (MCNP) simulation software [28] was used to evaluate the radiation attenuation characteristics of selected implant materials. Visual representations were generated to enhance interpretability, and the simulation outcomes were compared with theoretical calculations obtained via XCOM [29].

## 2. Materials and Methods

### 2.1. Study Design

**Figure 1:**
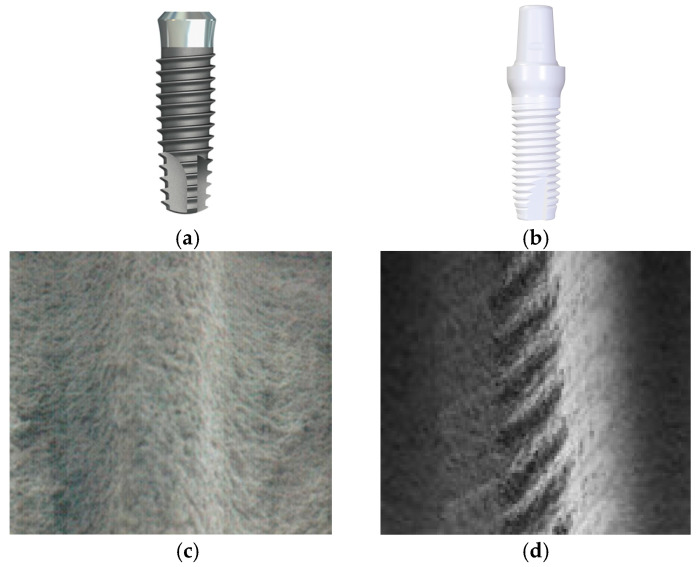
The shape and internal configurations of dental implants ((**a**–**c**): NTA titanium implant; (**b**–**d**): Z-Systems zirconia implant). The internal structures are shown in scanning electron microscope (SEM) images at 50× magnification. The NTA implant features (**c**) an SLA (sandblasted, large grit, acid-etched) surface, whereas the Z-Systems implant (**d**) possesses a sandblasted and laser-modified surface.

This study was approved by the Clinical Research Ethics Committee of the Faculty of Medicine at Akdeniz University and was conducted in accordance with the Declaration of Helsinki. Three different dental implant materials from distinct manufacturers were analyzed. Two were titanium-based implants (Bilimplant, Proimtech Health Products Inc., Istanbul, Turkey; NTA Implant, Pilatus Swiss Dental GmbH, Egolzwil, Switzerland), and the third was a 5Y-form zirconia implant (Z-Systems AG, Oensingen, Switzerland). The first titanium implant (NTA Implant) (Figure 1a,c) consisted of 90.39% titanium (Ti), 5.40% aluminum (Al), and 4.21% vanadium (V) by weight, with a density of 4.428 g/cm^3^. The second titanium implant (Bilimplant) was composed of 58% titanium (Ti), 33% oxygen (O), 7% iron (Fe), 1% carbon (C), and 1% nitrogen (N), with a density of 4.51 g/cm^3^. The third material evaluated was a TZP-A BIO-HIP zirconia implant (Z-Systems) (Figure 1b,d), consisting of 94.75% zirconium dioxide (ZrO_2_), 5.00% yttrium oxide (Y_2_O_3_), and 0.25% aluminum oxide (Al_2_O_3_). This zirconia implant had the highest density among the samples, measured at 6.05 g/cm^3^ (Table 1).

### 2.2. Analysis

This study utilized the MCNP6 simulation software to model the photon source characteristics, geometric configuration, radiation interactions with dental implant materials, and absorbed energy levels. Developed by Los Alamos National Laboratory, MCNP is a versatile radiation transport code capable of simulating the behavior of various particles within complex three-dimensional geometries [30]. It incorporates comprehensive physics models and extensive cross-section libraries, enabling accurate simulations across a wide energy spectrum.

For this research, simulations were conducted within an energy range relevant to dental radiology (50–100 kVp, 1–20 mA), ensuring that the modeled conditions closely reflect clinical imaging scenarios. Radiation attenuation was calculated using the Beer–Lambert Law [31], which describes the exponential decrease in γ-ray intensity as a function of material properties and thickness, as follows:

*I* = *I*_0_*e*^−^*^μx^*. (1)

The *μ* (cm^−^^1^) is the linear attenuation coefficient for a material, where *I* is the attenuated photon intensity and *I*_0_ is the unattenuated photon intensity; *x* (cm) is the thickness. The coefficient characterizing a given material is the density-independent, mass attenuation coefficient. The rearrangement of Equation (1) obtained the equation for the linear attenuation coefficient (LAC):

(2) $$\mu =\frac{1}{x}\;ln(I_{0}/I).$$

The mass attenuation coefficients (MACs) for the materials were derived from Equation (2) using the densities of the respective samples:

(3) $$\mu /\rho =\frac{1}{\rho x}\;ln(I_{0}/I).$$

The mass attenuation *μ*/*ρ* (cm^2^ g^−^^1^)—where *ρ* (g cm^−^^3^) is the measured density of the material, *I* is the attenuated photon intensity, *I*_0_ is the unattenuated photon intensity, and *x* (cm) is the thickness in Equation (3)—can be obtained from Equation (2) [16,17,18,31].

*MFP* refers to the average distance a photon travels within a material before undergoing an interaction, such as absorption or scattering. It is calculated as shown in Equation (4):

*MFP* = 1/*μ*. (4)

*TVL* refers to the thickness of a material required to reduce the intensity of a specific type of radiation to one-tenth of its original value. It is significantly influenced by the material’s density and elemental composition and can be calculated as shown in Equation (5):

(5) $$TVL=\frac{\ln10}{\mu }.$$

*TVL* is utilized to assess radiation absorption characteristics and comprehend a material’s capacity to absorb radiation. In this context, *TVL* values are often specified for X-rays or gamma rays at a particular energy, offering insights into the penetration depth of radiation into a material. It is utilized in applications including radiological safety and dose planning.

*HVL* refers to the thickness of a material required to reduce the intensity of a radiation beam to half of its original value. This value is directly influenced by the material’s density and elemental composition and is calculated as shown in Equation (6):

*HVL* = *ln*2/*μ*. (6)

These parameters are attenuation characteristics that are frequently employed in disciplines such as radiation physics and imaging technology. The optimization of radiation dose delivered to the patient and the improvement of image quality are both essential for the accurate diagnosis and effective treatment planning. Each parameter plays an important role in influencing these objectives.

**Figure 2:**
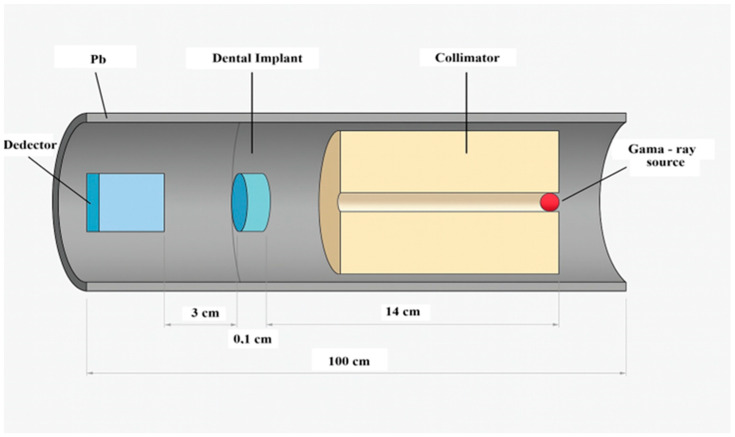
Schematic representation of the MCNP simulation geometry, including the photon source, implant, detector, and lead shielding used in a narrow-beam setup.

In order to investigate these attenuation characteristics quantitatively and assess their implications for dental imaging, an MC simulation was performed using MCNP6. Figure 2 shows the detailed geometry of the simulation setup. A simple narrow-beam geometry was constructed, consisting of a point gamma-ray source, a dental implant material slab, and a photon detector aligned along the beam axis. The geometry setup, illustrated in Figure 2, included lead shielding around both the source and the detector to minimize scattered radiation. The detector was designed to record the photon fluence in units of MeV^−^^1^·cm^−^^2^·s^−^^1^.

The MCNP6 code utilized the F4 tally to estimate the average photon flux passing through the implant material. Simulations were conducted with 10^7^ particle histories to ensure statistical accuracy. The recorded photon flux values were analyzed using the Lambert–Beer law to determine the linear attenuation coefficients (μ) for each material at selected photon energies.

## 3. Results

The MAC and LAC values of three different dental implant types (NTA Implant, Bilimplant, and Z-Systems) were calculated using both MCNP6 simulations and XCOM data across a diagnostic photon energy range of 50–100 keV. The results are presented in Table 2 and Table 3. The MAC analysis revealed that the zirconia-based Z-Systems implant exhibited significantly higher attenuation at all energy levels compared to the titanium-based NTA and Bilimplant. At 50 keV, the MAC of the Z-Systems implant reached 4.613 cm^2^/g (XCOM), whereas the values for the NTA implant and Bilimplant were 1.173 cm^2^/g and 0.915 cm^2^/g, respectively. This trend persisted across higher energies, underscoring the substantial influence of elemental composition and material density on photon absorption. To contextualize these findings, the calculated MAC values were compared with those of natural dental tissues reported in previous studies [32]. All implant materials demonstrated considerably higher MAC values than native dental tissues (e.g., ~0.35 cm^2^/g at 59.5 keV), suggesting a potential for contrast-related imaging artifacts when implants are in proximity to natural structures. The LAC results mirrored the MAC trends, with Z-Systems showing the highest LAC, peaking at 27.91 cm^−^^1^ at 50 keV, substantially exceeding those of NTA (5.19 cm^−^^1^) and Bilimplant (0.28 cm^−^^1^). As photon energy increased, LAC values declined for all materials, with the steepest drop observed in the zirconia implant. These results emphasize zirconia’s high photon attenuation capacity, which may enhance image contrast at higher energies but also increase the risk of beam-hardening artifacts in low-energy imaging modalities.

The relative deviation (%R.D.) between MCNP6 and XCOM results remained below 1% for all implant types and energy levels, demonstrating high consistency and mutual validation between both computational approaches.

**Table 2:** Comparison of mass attenuation coefficients (MACs) (cm^2^/g) for dental implants and teeth at diagnostic X-ray energies.

Energy (keV)	NTA Implant (cm^2^ g^−1^)			Bilimplant (cm^2^ g^−1^)			Z-Systems (cm^2^ g^−1^)			Teeth [32]
	XCOM	MCNP6	%R.D	XCOM	MCNP6	%R.D	XCOM	MCNP6	%R.D	
50	1.173	1.173	0.033	0.915	0.915	0.046	4.613	4.615	0.034	
54	0.963	0.964	0.039	0.759	0.760	0.064	3.742	3.746	0.100	
59.5	0.758	0.758	0	0.606	0.606	0.132	2.877	2.880	0.104	0.35 ± 0.02
60	0.743	0.743	0.030	0.595	0.594	0.154	2.813	2.816	0.101	
64	0.638	0.638	0.053	0.517	0.517	0.068	2.365	2.367	0.088	
70	0.522	0.521	0.069	0.430	0.430	0.027	1.863	1.865	0.087	
75	0.451	0.450	0.146	0.377	0.377	0.062	1.554	1.554	0.019	
80	0.396	0.396	0.069	0.335	0.335	0.045	1.314	1.314	0.032	
81	0.386	0.386	0.047	0.328	0.320	0.002	1.273	1.273	0.010	0.22 ± 0.01
84	0.360	0.360	0.027	0.309	0.309	0.021	1.160	1.160	0.003	
90	0.318	0.318	0.032	0.277	0.277	0.077	0.975	0.974	0.116	
95	0.290	0.290	0.039	0.256	0.256	0.273	0.853	0.852	0.125	
100	0.267	0.268	0.306	0.238	0.239	0.368	0.753	0.753	0.078	

**Table 3:** Linear attenuation coefficients (LACs) (cm^−^^1^) of dental implants at diagnostic X-ray energies.

Energy (keV)	NTA Implant (cm^−1^)			Bilimplant (cm^−1^)			Z-Systems (cm^−1^)		
	XCOM	MCNP6	%R.D	XCOM	MCNP6	%R.D	XCOM	MCNP6	%R.D
50	5.194	5.194	0	0.278	0.280	0.883	27.909	27.918	0.034
54	4.264	4.269	0.104	0.228	0.229	0.724	22.639	22.662	0.100
59.5	3.357	3.357	0	2.735	2.731	0.132	17.406	17.424	0.104
60	3.290	3.290	0	0.200	0.202	0.699	17.019	17.036	0.101
64	2.825	2.825	0	0.183	0.185	0.774	14.308	14.321	0.088
70	2.311	2.307	0.192	0.172	0.173	0.798	11.271	11.281	0.087
75	1.997	1.993	0.222	0.163	0.164	0.791	9.402	9.403	0.019
80	1.753	1.753	0	0.156	0.157	0.634	7.950	7.952	0.032
81	1.710	1.710	0.046	1.480	1.480	0.002	7.702	7.701	0.010
84	1.594	1.594	0	0.151	0.152	0.644	7.018	7.018	0.003
90	1.408	1.408	0	0.146	0.147	0.637	5.897	5.890	0.116
95	1.284	1.284	0	0.142	0.143	0.768	5.160	5.154	0.125
100	1.182	1.187	0	0.138	0.139	0.824	4.557	4.553	0.078

**Figure 3:**
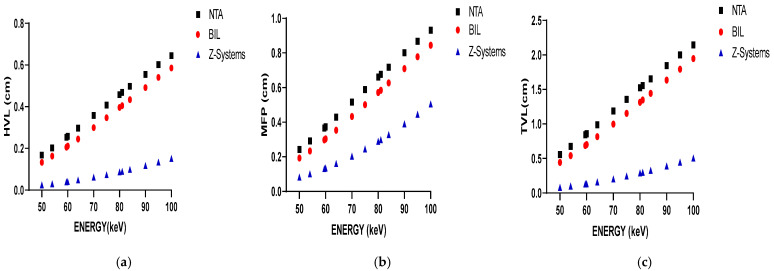
The variation in HVL results with photon energy (**a**); The variation in MFP results with photon energy (**b**); The variation in TVL results with photon energy (**c**).

Figure 3 illustrates the variation in HVL, MFP, and TVL values with increasing photon energy in parts (a), (b), and (c), respectively. Lower HVL values indicate that titanium alloys are more effective at attenuating low-energy photons, making them suitable for dental imaging modalities such as CBCT and panoramic radiography. However, their moderate density may contribute to image artifacts at higher energy levels. As shown in Figure 3a, HVL values increase with rising photon energy for all materials, reflecting a decline in attenuation efficiency at higher energies (e.g., in CT imaging).

In Figure 3b, it is observed that the MFP values for all materials increase with rising photon energy. This trend indicates that high-energy photons can travel greater distances within a material, thereby diminishing the material’s attenuation capability. Titanium alloys, which exhibit lower TVL values, are particularly effective at attenuating low-energy radiation, making them well suited for conventional dental radiology applications. However, due to their moderate density, titanium alloys may introduce imaging artifacts when used in conjunction with higher-energy radiation. Conversely, zirconium demonstrates higher TVL values, indicating superior attenuation efficiency in high-energy imaging contexts. As shown in Figure 3c, zirconium consistently exhibits the highest attenuation performance. For all materials analyzed, the TVL values increase with photon energy. Zirconium implants display lower MFP, HVL, and TVL values overall, suggesting that the X-ray beam is less penetrating. While this enhanced attenuation may be advantageous in certain scenarios, it can also result in more pronounced beam-hardening and streak artifacts in low-energy imaging modalities such as CBCT and panoramic radiography. These artifacts have the potential to obscure adjacent anatomical structures and complicate clinical interpretation. In contrast, titanium implants are more appropriate for use in low-energy imaging systems. However, their application in high-energy imaging modalities, such as CT, may lead to increased image artifacts, thereby impacting diagnostic accuracy.

## 4. Discussion

This study investigated the radiological behavior of various dental implant materials, with particular emphasis on the influence of material density and elemental composition on radiation attenuation properties. MC simulations using MCNP6, along with theoretical calculations based on the XCOM database, were employed to evaluate key parameters such as the HVL, MFP, and TVL across diagnostic photon energy levels relevant to dental imaging (50–100 keV).

The results indicate that titanium-based implants (NTA and Bilimplant) exhibit lower HVL and TVL values at lower energy levels, highlighting their potential suitability for low-energy imaging modalities such as CBCT and panoramic radiography. Their relatively low atomic number and mass contribute to a reduced likelihood of beam-hardening and streak artifacts—an important consideration for maintaining high image quality in clinical settings. Nevertheless, due to their moderate density, titanium alloys may still introduce artifacts when used in higher-energy imaging systems, such as conventional CT. On the other hand, zirconia-based implants (Z-Systems), which are characterized by a high density and atomic number, exhibited the highest MAC and LAC values among all the materials evaluated. These attributes render zirconia highly effective for high-energy applications due to its superior attenuation capacity. Nonetheless, these same properties can lead to excessive attenuation at lower energy levels, potentially causing beam-hardening artifacts and image distortions.

As demonstrated in Figure 3a–c, an increase in photon energy corresponds to higher HVL, MFP, and TVL values across all investigated materials. This trend reflects the reduced probability of photon–matter interactions at higher energies. While titanium implants offer sufficient attenuation at lower energies, zirconia exhibits superior performance under high-energy conditions. These results are consistent with previous studies, which have similarly reported increased artifact formation with zirconia implants and greater transmission with titanium implants, particularly at elevated kVp settings [12,13,33,34].

The simulation results also underscore the influence of structural factors on radiation attenuation. Titanium implants may exhibit porous microstructures [35], which can affect internal scattering and radiation focusing. In contrast, zirconia’s dense and homogeneous composition [36] facilitates more predictable interactions with the radiation beam. These intrinsic structural differences contribute to material-specific imaging outcomes. The presence of voids or pores [37] may lead to localized scattering or concentration of radiation, potentially impacting artifact formation. Radiation attenuation is further modulated by parameters such as material density, thickness, atomic number, and photon energy [38]. This is corroborated by the MAC and LAC values presented in Table 2 and Table 3. Zirconia implants consistently demonstrated higher attenuation coefficients than titanium implants across all diagnostic photon energy levels. Furthermore, all implant materials exhibited greater attenuation than natural dental tissue (e.g., ~0.35 cm^2^/g at 59.5 keV [32]), suggesting that image contrast discrepancies and diagnostic challenges may arise when implants are placed adjacent to native anatomical structures.

Despite the strengths of this computational investigation, several limitations should be acknowledged. First, all simulations were conducted under idealized and geometrically uniform conditions, which do not fully capture the anatomical complexity encountered in clinical scenarios. Second, this study lacks validation through experimental data or clinical imaging, which limits the direct translatability of the findings. Third, surface treatments of the implant materials were not considered. While this limitation has been explicitly acknowledged, it is important to emphasize that surface topography may influence radiation attenuation. Variations in surface roughness or treatment methods such as sandblasting, acid etching, or laser modification could alter the interaction between radiation and the implant material [39]. Future research should therefore explore the impact of different surface topographies on radiation attenuation to enhance our understanding of their role in imaging artifacts and diagnostic accuracy. Moreover, although two titanium implants and one zirconia implant were analyzed, only a single representative sample was used for each material type. This approach may overlook potential batch-to-batch variations or microstructural inconsistencies within the same material group. As such, caution should be exercised when generalizing the results, and future studies should incorporate multiple samples per implant type to ensure greater representativeness and reliability. These factors may affect the generalizability of the findings. Nevertheless, Monte Carlo-based simulations remain a robust tool for modeling complex interactions between radiation and materials in the absence of physical experimentation. By systematically varying parameters such as material thickness, density, and structure, MCNP6 enables detailed and reproducible analyses. The strong concordance observed between MCNP6 and XCOM across all coefficients (with a relative deviation of less than 1%) further supports the validity of the methodology.

This study provides a comprehensive evaluation of the impact of different dental implant materials on X-ray attenuation across various diagnostic imaging modalities. The findings underscore the importance of material selection in relation to the imaging system’s energy spectrum, demonstrating that appropriate material choices can substantially minimize imaging artifacts and enhance diagnostic accuracy.

## 5. Conclusions

This study comprehensively evaluated the radiation attenuation characteristics of dental implant materials, namely titanium alloys and zirconia, using Monte Carlo simulation methods. The results emphasize that the interaction of implant materials with radiation, especially regarding image quality and artifact formation, should be taken into account in clinical decision-making.

Titanium alloys, owing to their moderate density and atomic number, are more suitable for low-to-medium energy imaging systems such as CBCT and panoramic radiography. They offer sufficient photon attenuation while minimizing image artifacts in this energy range. However, in high-energy modalities (e.g., CT), they may lead to increased artifacts. Conversely, zirconia implants provide superior attenuation in high-energy systems due to their higher density and atomic number but can induce beam hardening and streak artifacts in low-energy imaging.

Future research should focus on experimental validation of these simulation results and their correlation with clinical imaging findings. Such studies would reinforce the clinical applicability of these findings and demonstrate how implant material selection contributes to both image quality and patient safety.

## Figures and Tables

**Table 1:** The elemental composition and density ratios of dental implants.

Implant	Element	Weight (%)	Implant Density (g/cm^3^)
NTA Implant	Titanium (Ti)	90.39	4.428
	Aluminum (Al)	5.4	
	Vanadium (V)	4.21	
Bilimplant	Carbon (C)	1.00	4.510
	Nitrogen (N)	1.00	
	Iron (FE)	7.00	
	Oxygen (O)	33.00	
	Titanium (Ti)	58.00	
Z-Systems	Zirconia (ZrO_2_)	94.75	6.05
	Yttrium Oxide (Y_2_O_3_)	5.00	
	Aluminum Oxide (Al_2_O_3_)	0.25	

## Data Availability

The data supporting the findings of this study can be provided upon reasonable request to the corresponding author, S.T.

## Abbreviations

CT	Computed tomography
CBCT	Cone-beam computed tomography
Ti	Titanium
MC	Monte Carlo
MCNP	Monte Carlo N-particle
MFP	Mean free path
TVL	Tenth-value layer
HVL	Half-value layer
ZiO_2_	Zirconium dioxide
Al_2_O_3_	Aluminum oxide
Y_2_O_3_	Yttrium oxide
FOV	Field of view

## References

[1] Steigenga J.T., Al-Shammari K.F., Nociti F.H., Misch C.E., Wang H.L. (2003). Dental Implant Design and Its Relationship to Long-Term Implant Success. Implant. Dent..

[2] Schiegnitz E., Müller L.K., Sagheb K., Theis L., Cagiran V., Kämmerer P.W., Wegener J., Wagner W., Al-Nawas B. (2021). Clinical Long-Term and Patient-Reported Outcomes of Dental Implants in Oral Cancer Patients. Int. J. Implant. Dent..

[3] Adell R. (1981). A 15-Year Study of Osseointegrated Implants in the Treatment of the Edentulous Jaw. Int. J. Oral Surg..

[4] Van Oirschot B.A.J.A., Zhang Y., Alghamdi H.S., Cordeiro J.M., Nagay B.E., Barao V.A.R., De Avila E.D., Van Den Beucken J.J.J.P. (2022). Surface Engineering for Dental Implantology: Favoring Tissue Responses Along the Implant. Tissue Eng. Part A.

[5] Thanissorn C., Guo J., Jing Ying Chan D., Koyi B., Kujan O., Khzam N., Miranda L.A. (2022). Success Rates and Complications Associated with Single Immediate Implants: A Systematic Review. Dent. J..

[6] Kirsch A., Ackermann K.L. (1989). The IMZ Osteointegrated Implant System. Dent. Clin. N. Am..

[7] Buser D., Sennerby L., De Bruyn H. (2017). Modern Implant Dentistry Based on Osseointegration: 50 Years of Progress, Current Trends and Open Questions. Periodontol. 2000.

[8] Cionca N., Hashim D., Mombelli A. (2017). Zirconia Dental Implants: Where Are We Now, and Where Are We Heading?. Periodontol. 2000.

[9] Schulze R. (2022). CBCT Artefact-Burden of Zirconia-Based as Compared to Titanium Implants for Different Beam Energies: An Analytical Approach. Sci. Rep..

[10] Salian S.S., Subhadarsanee C.P., Patil R.T., Dhadse P. (2024). V Radiographic Evaluation in Implant Patients: A Review. Cureus.

[11] Almulhim S.A.N., Busaeed A.R.A., Alhaji Y.B., Sallam J.A., Aljuaid A.S.E., Eishan A.A.M., Alharbi M.A., Shehatah A.U., Asiri M.H.M., al Baqshi H.A. (2022). Comparative study of different imaging modalities in assessing dental implant stability. J. Popul. Ther. Clin. Pharmacol..

[12] Freitas D.Q., Fontenele R.C., Nascimento E.H.L., Vasconcelos T.V., Noujeim M. (2018). Influence of Acquisition Parameters on the Magnitude of Cone Beam Computed Tomography Artifacts. Dentomaxillofac. Radiol..

[13] Schulze R., Heil U., Groß D., Bruellmann D.D., Dranischnikow E., Schwanecke U., Schoemer E. (2011). Artefacts in CBCT: A Review. Dentomaxillofac. Radiol..

[14] Kovalchuk O., Ponton A., Filkowski J., Kovalchuk I. (2004). Dissimilar Genome Response to Acute and Chronic Low-Dose Radiation in Male and Female Mice. Mutat. Res. Mol. Mech. Mutagen..

[15] Van Gompel G., Van Slambrouck K., Defrise M., Batenburg K.J., De Mey J., Sijbers J., Nuyts J. (2011). Iterative Correction of Beam Hardening Artifacts in CT. Med. Phys..

[16] Abdel-Rahman M.A., Badawi E.A., Abdel-Hady Y.L., Kamel N. (2000). Effect of Sample Thickness on the Measured Mass Attenuation Coefficients of Some Compounds and Elements for 59.54, 661.6 and 1332.5 KeV γ-Rays. Nucl. Instrum. Methods Phys. Res. A.

[17] Singh K., Kaur G., Sandhu G.K., Lark B.S. (2001). Interaction of Photons with Some Solutions. Radiat. Phys. Chem..

[18] Shakhreet B.Z., Chong C.S., Bandyopadhyay T., Bradley D.A., Tajuddin A.A., Shukri A. (2003). Measurement of Photon Mass-Energy Absorption Coefficients of Paraffin Wax and Gypsum at 662 KeV. Radiat. Phys. Chem..

[19] Ozdogan H., Kilicoglu O., Akman F., Agar O. (2022). Comparison of Monte Carlo Simulations and Theoretical Calculations of Nuclear Shielding Characteristics of Various Borate Glasses Including Bi, V., Fe, and Cd. Appl. Radiat. Isot..

[20] Akman F., Kilicoglu O., Agar O. (2023). Feasibility of a Novel Shield of Nuclear Radiation with W–Ni–Fe–Co and La–Bi Alloys Alternative to Pb and Ordinary Concrete Absorbers. Prog. Nucl. Energy.

[21] Tousi E.T. (2022). Monte Carlo Simulation of the Mass Attenuation Coefficient and Effective Atomic Number of the Eremurus-Rhizophora Ssp. Particleboard Phantom at the Mammography Energy Range. Prog. Nucl. Energy.

[22] Lee H. (2024). Monte Carlo Methods for Medical Imaging Research. Biomed. Eng. Lett..

[23] Saidi P., Sadeghi M., Tenreiro C., Saidi P., Sadeghi M., Tenreiro C. (2013). Variance Reduction of Monte Carlo Simulation in Nuclear Engineering Field. Theory and Applications of Monte Carlo Simulations.

[24] Metropolis Monte Carlo and The Maniac | PDF | Physical Sciences. https://mcnp.lanl.gov/pdf_files/Article_1986_LAS_Anderson_96--108.pdf.

[25] Harrison R.L. (2010). Introduction to Monte Carlo Simulation. AIP Conf. Proc..

[26] Boone J.M., McNitt-Gray M.F., Hernandez A.M. (2017). Monte Carlo Basics for Radiation Dose Assessment in Diagnostic Radiology. J. Am. Coll. Radiol..

[27] Andreo P. (1991). Monte Carlo Techniques in Medical Radiation Physics. Phys. Med. Biol..

[28] Goorley J.T., James M.R., Booth T.E., Brown F.B., Bull J.S., Cox L.J., Durkee J.W., Elson J.S., Fensin M.L., Forster R.A. (2013). Initial MCNP6 Release Overview-MCNP6 Version 1.0.

[29] Gerward L., Guilbert N., Jensen K.B., Levring H. (2004). WinXCom—A Program for Calculating X-Ray Attenuation Coefficients. Radiat. Phys. Chem..

[30] Stults K.A., James M.R., Lombardi M., Klasky M.L. (2020). Comparison of Modeled to Measured Spectra Using MCNP and GADRAS to Benchmark and Contrast Modeling Limitations.

[31] Bashter I.I. (1997). Calculation of Radiation Attenuation Coefficients for Shielding Concretes. Ann. Nucl. Energy.

[32] Kurudirek M., Topcuoglu S. (2011). Investigation of Human Teeth with Respect to the Photon Interaction, Energy Absorption and Buildup Factor. Nucl. Instrum. Methods Phys. Res. B.

[33] Sancho-Puchades M., Hämmerle C.H.F., Benic G.I. (2015). In Vitro Assessment of Artifacts Induced by Titanium, Titanium-Zirconium and Zirconium Dioxide Implants in Cone-Beam Computed Tomography. Clin. Oral. Implant. Res..

[34] Shokri A., Vafaee F., Haghighat L., Shahabi S., Farhadian M., Jamalpour M.R. (2022). Comparison of the Amount of Artifacts Induced by Zirconium and Titanium Implants in Cone-Beam Computed Tomography Images. BMC Med. Imaging.

[35] Huang C.C., Li M.J., Tsai P.I., Kung P.C., Chen S.Y., Sun J.S., Tsou N.T. (2020). Novel Design of Additive Manufactured Hollow Porous Implants. Dent. Mater..

[36] Tchinda A., Lerebours A., Kouitat-Njiwa R., Bravetti P. (2023). Zirconia Dental Implants: A Closer Look at Surface Condition and Intrinsic Composition by SEM-EDX. Bioengineering.

[37] Choudhury N.R.R., Vilaplana R., Botet R., Sen A.K. (2020). Comparison of Light Scattering Properties of Porous Dust Particle with Connected and Unconnected Dipoles. Planet. Space Sci..

[38] Halliwell E., Couch C., Begum R., Li W., Maqbool M. (2021). Increase in Linear Attenuation Coefficient by Changing Crystal Structure of Materials for Radiation Shielding and Biomedical Devices Safety. Colloids Surf. A Physicochem. Eng. Asp..

[39] Assessment of Radiation Attenuation Properties in Dental Implants Using Monte Carlo Method. Proceedings of the 35th International Congress of Oral Diagnosis and Maxillofacial Radiology Association.

